# Anti-oxidant copper layer by remote mode N_2_ plasma for low temperature copper–copper bonding

**DOI:** 10.1038/s41598-020-78396-x

**Published:** 2020-12-10

**Authors:** Haesung Park, Hankyeol Seo, Sarah Eunkyung Kim

**Affiliations:** 1 Department of Mechanical Engineering, Seoul National University of Science and Technology, 232 Gongneung-ro, Nowon-gu, Seoul, 01811 Korea; 2 Graduate School of Nano-IT Design, Seoul National University of Science and Technology, 232 Gongneung-ro, Nowon-gu, Seoul, 01811 Korea

**Keywords:** Engineering, Materials science

## Abstract

An anti-oxidant Cu layer was achieved by remote mode N_2_ plasma. Remote mode plasma treatment offers the advantages of having no defect formation, such as pinholes, by energetic ions. In this study, an activated Cu surface by Ar plasma chemically reacted with N free radicals to evenly form Cu nitride passivation over the entire Cu surface. According to chemical state analysis using XPS, Cu oxidation was effectively prevented in air, and the thickness of the Cu nitride passivation was within 3 nm. Based on statistical analysis using the DOE technique with N_2_ plasma variables, namely, RF power, working pressure, and plasma treatment time, we experimentally demonstrated that a lower RF power is the most effective for forming uniform Cu nitride passivation because of a lower plasma density. When the N_2_ plasma density reached approximately 10^9^ cm^−3^ in which the remote mode was generated, high energy electrons in the plasma were significantly reduced and the amount of oxygen detected on the Cu surface was minimized. Finally, low temperature (300 °C) Cu–Cu bonding was performed with a pair of the anti-oxidant Cu layers formed by the remote mode N_2_ plasma. Cu atomic diffusion with new grains was observed across the bonded interface indicating significantly improved bonding quality over bare Cu–Cu bonding.

## Introduction

In advanced packaging such as 3D packaging that vertically stacks the manufactured chips and heterogeneous packaging that places various chips in a limited area, Cu–Cu bonding as electrical connections between the chips is a key process for the high performance and reliability of the entire system^1–10^. In order to protect a device from thermal damage in device stacking, a low temperature (≤ 300 °C) Cu–Cu bonding process is essential. However, low temperature Cu–Cu bonding is interrupted by the native oxide film such as Cu_2_O which blocks Cu atomic diffusion at the Cu–Cu bonding interface^11,12^. Thus, Cu–Cu bonding generally requires a bonding temperature higher than 400 °C to break Cu oxide layer.

Many Cu–Cu bonding studies have been reported on lowering the bonding temperature. One such approach is removing the native oxide film that has already formed on a Cu surface. This approach includes a surface activated bonding (SAB) method^13,14^ that breaks the Cu oxide layer and activates the Cu surface using collision from a high energy Ar beam and a wet treatment method^15,16^ that chemically removes the Cu oxide layer using various acids, such as sulfuric acid (H_2_SO_4_), acetic acid (CH_3_COOH), and citric acid (C_6_H_8_O_7_). Alternatively, there is a way to prevent Cu oxidation from the beginning. Temporary passivation using a self-assembled monolayer (SAM) of alkane-thiol^17,18^ and metal passivation using Ti, Pd, Ag, or Au^19,20^ allow the Cu surface to remain pristine.

For high-volume manufacturing (HVM), Cu-Sn alloys have been applied to eutectic bonding thanks to its low melting point with a reasonable electrical conductivity^21,22^. Although Sn is used as a cap on the top of a Cu pillar bump, it is not suitable for a fine pitch (≤ 10 μm) process due to the reflow characteristics. In addition, the formation of intermetallic compounds (IMC) and the growth of Kirkendall void at the bonding interface degrade mechanical and electrical properties of the Cu/Sn/Cu interconnects. The Direct Bond Interconnect (DBI) technology^23,24^ is currently used in low temperature hybrid bonding applications, for example, in CMOS image sensors (CIS). That technology forms oxide-oxide bonding, and then a uniform shallow Cu recess is annealed at a low temperature (150–300 °C) to establish a Cu–Cu connection using the difference in the coefficient of thermal expansion between the oxide and Cu. However, that method requires a highly accurate chemical mechanical polishing (CMP) process control, and it is hard to apply it to ultra-fine pitch for the next generation.

With this background, we propose a passivation process using a Cu nitride layer in a low temperature Cu–Cu bonding method. This technique has numerous advantages compared to the other methods introduced above. First, our passivation process is CMOS compatible because it is a dry process that uses plasma treatment. It is also inexpensive and simple because the process is performed with a conventional DC sputter used for the Cu thin film deposition by common gases such as argon and nitrogen. Moreover, Cu nitride passivation protects the Cu surface from oxidation in the atmosphere, but it is easily decomposed at a low bonding temperature because of its thermodynamic instability. This characteristic helps to form a homogeneous bonded layer in a low temperature Cu–Cu bonding process.

In this study, the Cu nitride passivation was formed using a two-step Ar/N_2_ plasma process. Because the Cu nitride passivation can be damaged from the accompanying sputtering effect by Ar ion bombardment, the Ar + N_2_ mixed gas is unfavorable to the formation of uniform and thin copper nitride passivation in manufacturing. Therefore, we propose the two-step Ar/N_2_ plasma process: Ar plasma pre-treatment was performed first on a Cu surface to remove any contaminants and to activate Cu atoms before the N_2_ plasma treatment. Then, the N free radicals generated by the dissociation of N_2_ molecules in the N_2_ plasma chemically react with the activated Cu atoms at the Cu surface to form Cu nitride passivation. We report the effect of our two-step Ar/N_2_ plasma process on the Cu surface, the mechanism of the formation of Cu nitride passivation, and the bonding quality of low temperature Cu–Cu bonding using an anti-oxidant Cu layer.

## Methods

### Sample fabrication flow and DOE setup

An 8-inch Si wafer with a 700 nm thick SiO_2_ layer was diced into a chip size of 1 cm^2^. Each piece of diced Si chip specimen was fixed by Kapton tape on an 8-inch dummy Si wafer. Using DC magnetron sputtering, a 50 nm thick Ti adhesion layer and 1 μm thick Cu thin film were deposited on the diced Si chip specimens under 5 mTorr working pressure and 2500 W DC power. After the Cu deposition, two-step Ar/N_2_ plasma treatment was performed in the same sputter chamber with maintained vacuum conditions. For the plasma treatment, RF power of 13.56 MHz was applied to a table chuck where the specimen was located, and it became a cathode. In this study, we focused on the effect of N_2_ plasma variables on the formation of Cu nitride passivation. Thus, we fixed the variables of the Ar plasma process while we evaluated the N_2_ plasma process under the statistically designed conditions. In the design of experiment (DOE) method, central composite design (CCD) was used for a response surface methodology (RSM) which is an efficient DOE method that estimates the regression coefficient of a quadratic polynomial model with only a few experiments^25,26^. A total of 20 experiments for the N_2_ plasma process consisted of 8 points from the 3-factor (RF power, working pressure, and treatment time) 2-level (low or high) full factorial design experiments, 6 axial points, and 6 center points. The detailed two-step Ar/N_2_ plasma process conditions are listed in Table 1 and a study on the Ar plasma process using the full factorial DOE method was reported in previous paper^27^.

**Table 1:** Independent factors and levels (coded and actual) for CCD design (top), fixed Ar plasma treatment conditions (middle), N_2_ plasma treatment conditions designed by CCD (bottom).

Coded factor	Actual factor	Coded level and actual level				
		− α	− 1	0	+ 1	+ α
A	RF power	25.68	70	135	200	244.32
B	Pressure	3.48	4.5	6	7.5	8.52
C	Time	31.82	100	200	300	368.18

Ar plasma treatment conditions						
Specimen	Flow rate (sccm)	RF power (W)	Pressure (mTorr)	Time (s)		
Step 1 (fixed)						
All	150	100	7.5	30		

N_2_ plasma treatment conditions						
Specimen	Flow rate (sccm)	RF power (W)	Pressure (mTorr)	Time (s)		
**Step 2**						
1	45	70	4.5	100		
2		200	4.5	100		
3		70	7.5	100		
4		200	7.5	100		
5		70	4.5	300		
6		200	4.5	300		
7		70	7.5	300		
8		200	7.5	300		
9		26	6.0	200		
10		244	6.0	200		
11		135	3.5	200		
12		135	8.5	200		
13		135	6.0	32		
14		135	6.0	368		
15		135	6.0	200		
16		135	6.0	200		
17		135	6.0	200		
18		135	6.0	200		
19		135	6.0	200		
20		135	6.0	200		

For the Cu–Cu bonding process, the above deposition procedure and two-step Ar/N_2_ plasma process were carried out on an 8-inch Si wafer. A pair of Cu blanket wafers were bonded at 300 °C under 700 kPa for 1 h using a thermo-compression bonding method in the SB 8e wafer bonder of SUSS Microtech.

### The chemical state of Cu surface and Cu–Cu bonding interface analysis methods

The chemical state of the two-step Ar/N_2_ plasma treated Cu surface was analyzed by X-ray photoelectron spectroscopy (XPS). The peak profiles for three elements, Cu, O, and N, were measured using a Thermo Scientific K-Alpha^+^ XPS system with a micro-focused monochromatic Al Kα (hν = 1486.6 eV) X-ray source of 72 W at 12 kV.

The bonding quality of the bonded wafer was evaluated using scanning acoustic tomography (SAT) of Hitachi FineSAT III with a 140 MHz probe. For a detailed analysis of the bonding interface, the bonded wafer was diced into 1 cm^2^ specimens and the diced plane was polished by Ar ion beam milling using JEOL Ltd. IB-19510CP. Then the polished clear cross section was analyzed by field emission scanning electron microscope (FE-SEM) using FEI Company Apreo S HiVac with 20,000 × magnifications.

## Results and discussion

### The chemical state analysis of the two-step Ar/N_2_ plasma treated Cu surface by XPS

**Figure 1:**
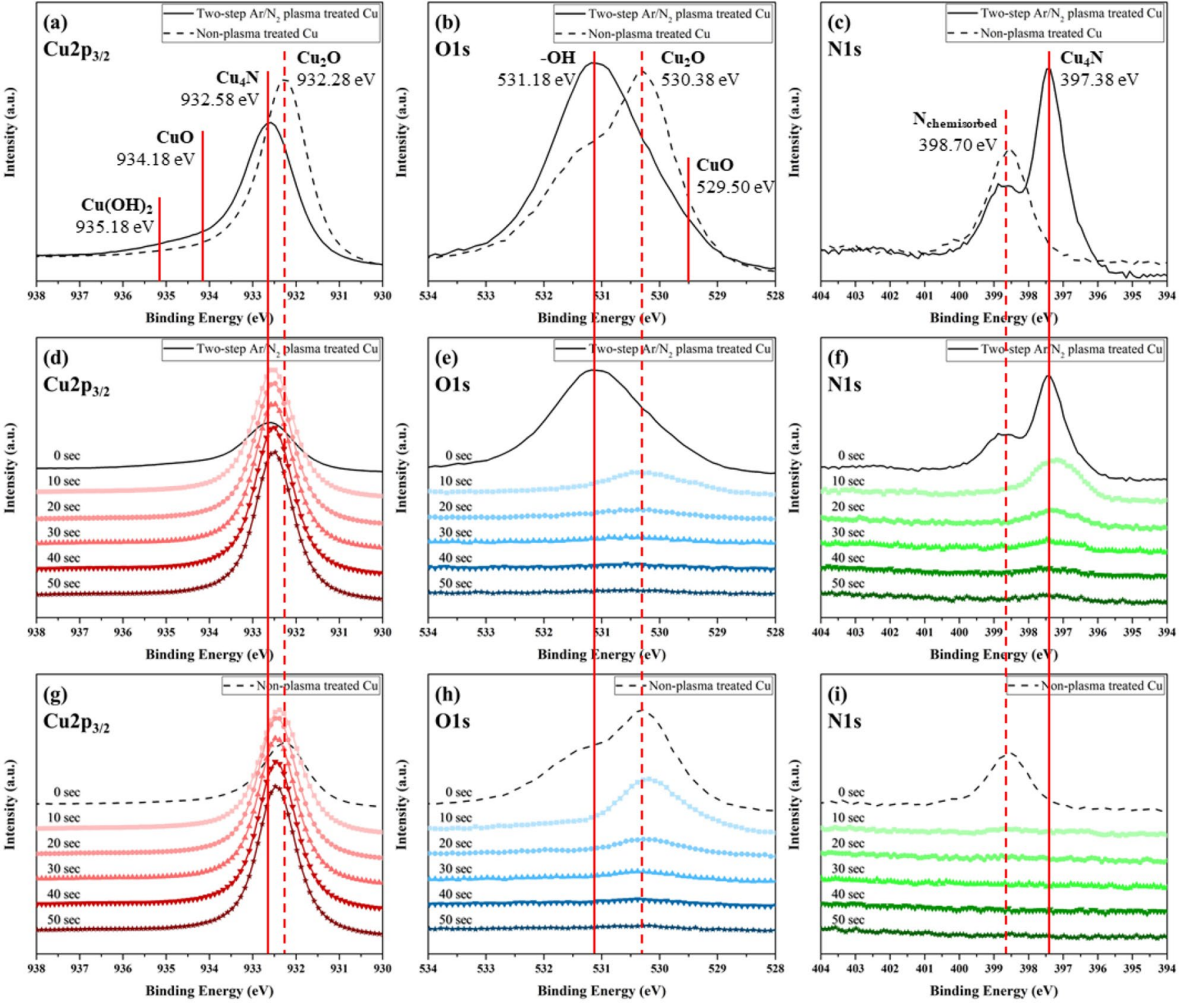
XPS analysis of Cu2p_3/2_, O1s, and N1s: (**a**–**c**) surface profiles of two-step Ar/N_2_ plasma treated Cu and non-plasma treated Cu, (**d**–**f**) depth profiles of two-step Ar/N_2_ plasma treated Cu and (**g**–**i**) depth profiles of non-plasma treated Cu for 50 s.

**Figure 2:**
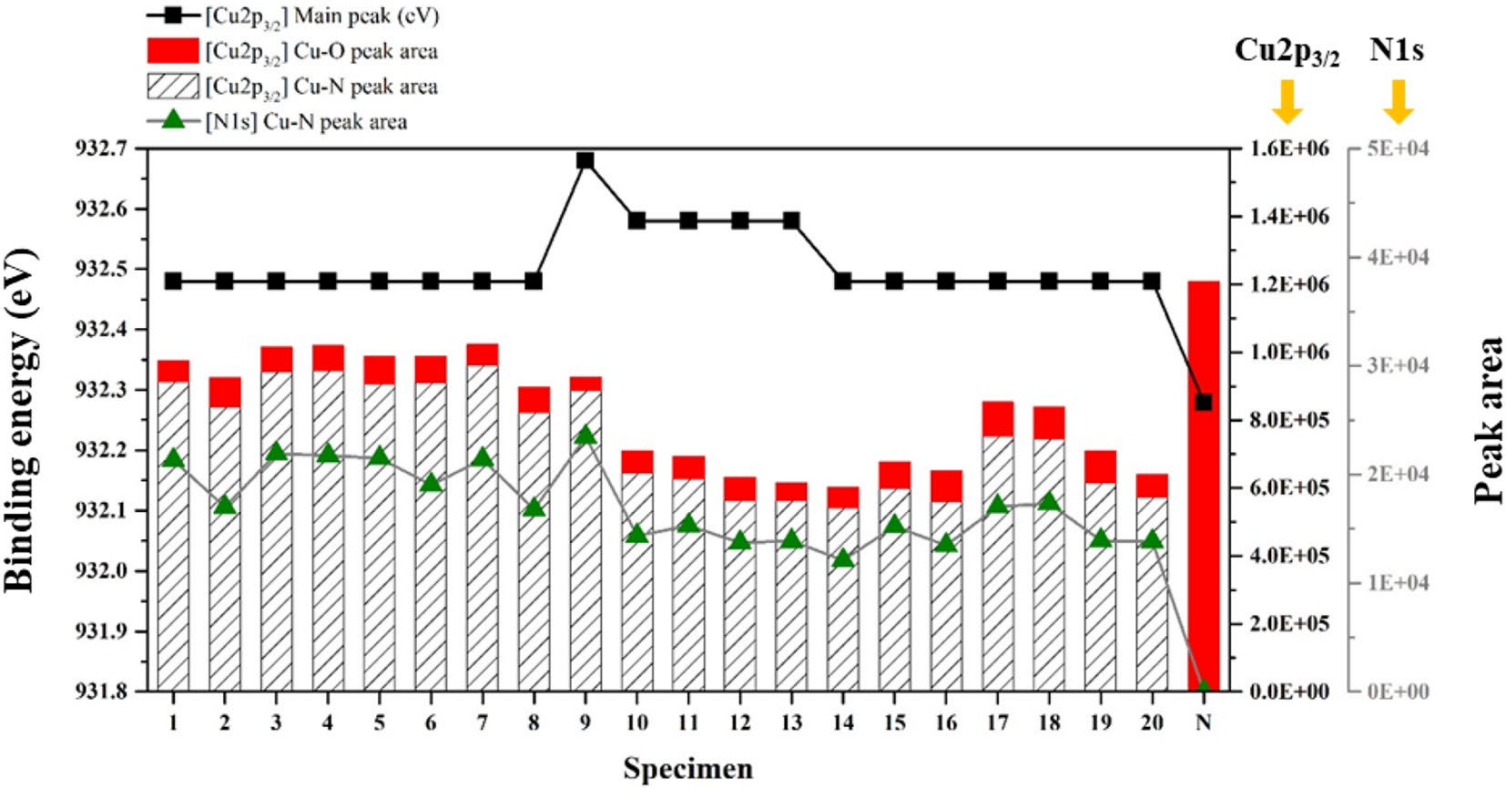
XPS peak analysis by a deconvolution technique.

Figures 1a–c compare the XPS profiles of the two-step Ar/N_2_ plasma treated Cu and non-plasma treated Cu, and Table 2 shows the reference binding energy of several Cu compounds^28–31^. After the plasma treatment, the main peak of the Cu2p_3/2_ profile in Fig. 1a shifted towards a higher binding energy (from 932.48 eV to 932.58 eV) indicating that a pure metallic Cu (Cu^0^) combined with other elements and new substances were created, namely, Cu nitride (Cu^1+^) or cupric oxide (Cu^2+^). On the other hand, the main peak of the non-plasma treated Cu shifted towards a lower binding energy (from 932.48 eV to 932.28 eV) than pure Cu due to the formation of cuprous oxide (Cu^1+^), which indicated oxidation in air. In addition, a noticeable peak at 530.38 eV of the O1s profile in Fig. 1b disappeared while a new peak at 397.38 eV of the N1s profile in Fig. 1c appeared. Therefore, the chemical states of the new peak of the N1s profile and the removed peak of O1s profile are considered as a Cu nitride layer and native oxide grown on Cu, respectively. The stoichiometry of each compound was estimated to be close to Cu_4_N and Cu_2_O by atomic concentration calculation (not shown here). In order to analyze the thickness of the Cu_4_N, XPS depth profiling was performed for 50 s at intervals of 10 s with an etch rate of 0.1 nm/sec. The Cu2p_3/2_ main peaks of the two-step Ar/N_2_ plasma treated Cu shown in Fig. 1d and the non-plasma treated Cu shown in Fig. 1g appeared at a binding energy value for pure Cu after 30 s. The Cu_4_N peak of the N1s profiles shown in Fig. 1f clearly appeared until 30 s of depth profiling, which indicated that the thickness of the Cu nitride was approximately 3 nm. In contrast, there is no peak in Fig. 1i after 10 s because the non-plasma treated Cu has only chemisorbed nitrogen atoms (N_chemisorbed_) on the Cu surface. The Cu_2_O peak of the O1s profile in non-plasma treated Cu shown in Fig. 1h was observed up to 30 s, which means that about 3 nm of native Cu oxide was formed without the plasma treatment. A peak of the O1s profile in the two-step Ar/N_2_ plasma treated Cu shown in Fig. 1e was observed until 10 s, however, it is considered as the peak due to chemisorbed oxygen atoms generated from a hydroxyl group (–OH) during depth profiling. Using the deconvolution technique, the binding energy, Cu–O peak area, and Cu–N peak area of the Cu2p_3/2_ profile and the Cu–N peak area of the N1s profile for all the specimens are plotted in Fig. 2. In the Cu2p_3/2_ profiles, all plasma treated Cu specimens have a Cu–N (Cu_4_N) peak area that is almost 10 times greater than its Cu–O (CuO) peak area. Moreover, Note that -OH peak area might be included in Cu–O peak area during the deconvolution analysis because the CuO peak is adjacent to the Cu(OH)_2_ peak within the narrow area of the Cu2p_3/2_ profile. Therefore, we considered the small area of Cu–O peak of the two-step Ar/N_2_ plasma treated Cu specimens to be negligible, and the surface of specimen 9 in particular to be an anti-oxidant Cu layer. In sharp contrast, the non-plasma treated Cu specimen had a significant amount of Cu–O (CuO + Cu_2_O) peak area. This was in agreement with the binding energy shift of the Cu2p_3/2_ main peak. These results prove that Cu oxide was effectively removed after the two-step Ar/N_2_ plasma treatment.

**Table 2:** Reference XPS binding energies of the Cu compounds^28–31^.

Linked to	Cu2p_3/2_ (eV)	O1s (eV)	N1s (eV)
Cu	932.0–932.5	–	–
Cu_2_O	932.2–932.8	529.9–530.6	–
CuO	933.6–934.4	529.4–529.8	–
–OH	935.0–935.3	~ 531.2	–
N_chemisorbed_	–	–	398.3–400.0
Cu_4_N	932.2–932.7	–	~ 397.4

### Effect of the N_2_ plasma variables on the formation of Cu nitride passivation

**Figure 3:**
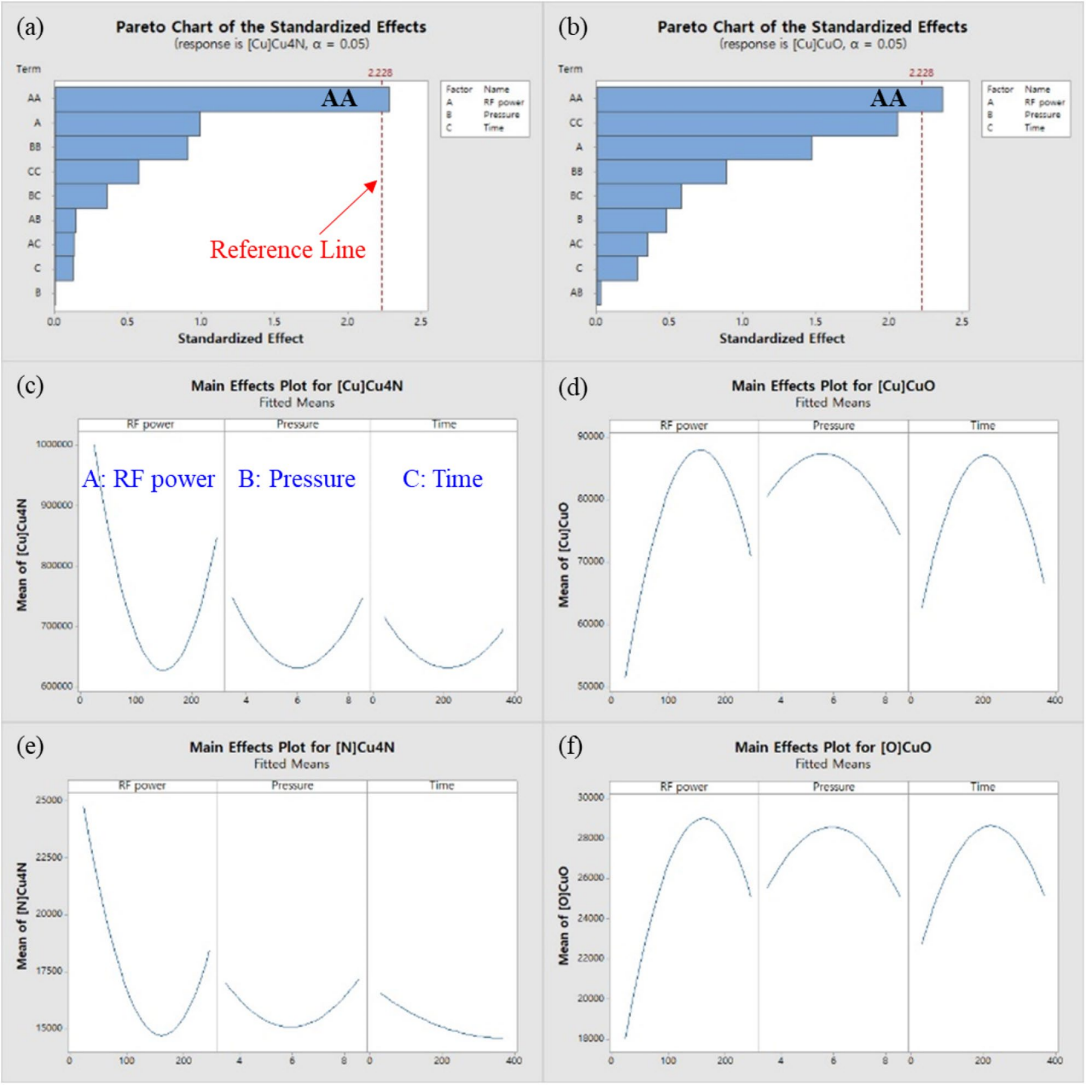
Analysis of the response surface design at 95% confidence level using a Minitab program (**a**) the Pareto chart of the standardized effects for the Cu_4_N peak of the Cu2p_3/2_, and the place where the term crosses the reference line is statistically significant, (**b**) the Pareto chart of the standardized effects for CuO peak of the Cu2p_3/2_, and (**c**-**f**) the main effects plot calculated by the response for Cu_4_N and CuO in the Cu2p_3/2_, O1s, and N1s.

To understand the effect of the N_2_ plasma variables on the formation of Cu nitride passivation, the correlation between the N_2_ plasma variables and the Cu_4_N peak area (also the CuO peak area just for comparison) of the Cu2p_3/2_ was statistically analyzed at 95% confidence (α = 0.05) using a Minitab program. The Pareto chart of the standardized effects and main effects plot for Cu_4_N and CuO are shown in Fig. 3a–d. Among the three variables (A: RF power, B: working pressure, and C: treatment time), the RF power (AA) is the most dominant factor for the Cu_4_N formation, and the working pressure and treatment time do not have much effect. AA is a quadratic term of the variable A, meaning that the variable and response (XPS peak area) have a nonlinear correlation. From the main effects plot, the lower RF power leads to more Cu_4_N formation, but meanwhile, the analysis results for the CuO are the opposite because CuO production is prevented by Cu_4_N. Additional analysis results for the Cu_4_N peak area of the N1s shown in Fig. 3e and the CuO peak area of the O1s shown in Fig. 3f coincide with those for Cu2p_3/2_ shown in Fig. 3c,d. The effect of RF power in N_2_ plasma treatment is explained in detail below.

### Effect of the RF power on the N_2_ plasma density

Depending on the applied RF power (P_RF_), electrons inside the plasma collide with molecules or atoms to transfer energy or dissipate into the wall. The power absorbed by electrons (P_abs_) is balanced with the power consumed by generation of electrons and ions through various collisions at a steady state (P_loss_). Therefore, if we ignore the power loss that occurred in the RF matching box, we can be assume that P_RF_ = P_abs_ = P_loss_, and the total power loss is as follows^32,33^.

1 $$ P_{loss} = n_{s} u_{B} Ae\varepsilon_{T} $$

where $n_{s}$, $u_{B}$, $A$, and $e\varepsilon_{T}$ represent the electron density at the sheath boundary, Bohm velocity (the sound speed for cold ions), interior area of the plasma chamber, and total loss energy required to generate an electron–ion pair, respectively. The $n_{s}$ has a relationship with the plasma density $n_{0}$ as $n_{s} = h\left( {\lambda_{i} } \right)n_{0}$ and where $\lambda_{i}$ is an ion mean free path, $h$ denotes the plasma density ratio to the radial ($h_{R}$) and axial ($h_{L}$) directions in the plasma chamber when the radius of the plasma chamber is R and the height is L. Equation (1) can be expressed as $P_{loss} = P_{RF} = h\left( {\lambda_{i} } \right)n_{0} u_{B} Ae\varepsilon_{T}$, where $h\left( {\lambda_{i} } \right)A = A_{eff} = 2\pi R^{2} h_{L} + 2\pi RLh_{R}$. Finally, the equation for the plasma density becomes^32,34^

2 $$ n_{0} = \frac{{P_{RF} }}{{u_{B} e\varepsilon_{T} A_{eff} }} $$

**Figure 4:**
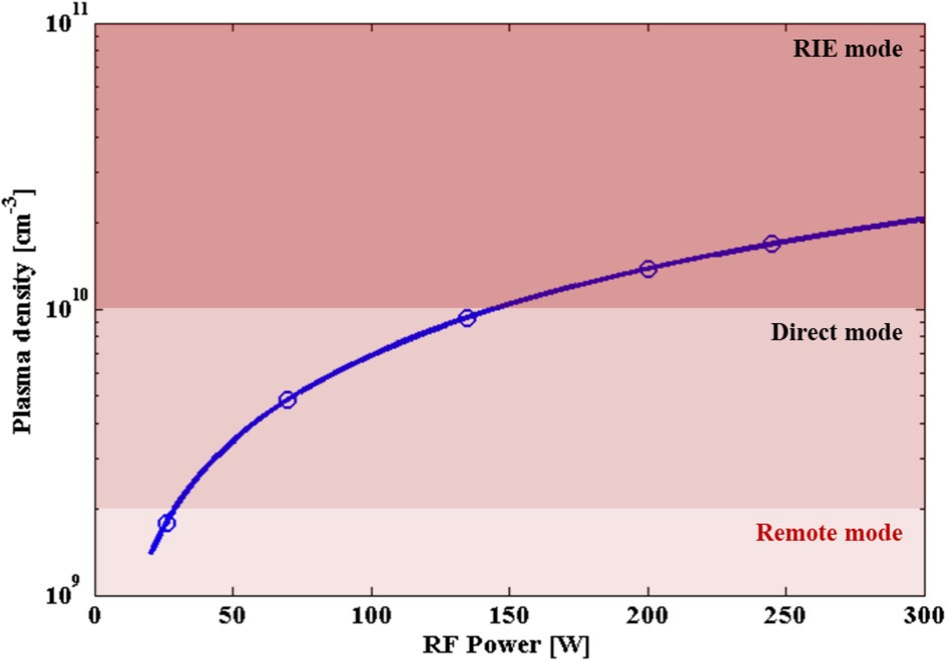
Plasma density and mode depending on the RF power.

The three terms of the denominator are a function of the electron temperature and are negligible for the plasma density within the designed experimental range in this research. On the other hand, the plasma density significantly increases in proportion to the RF power (26–244 W), and the calculated plasma density as a function of the RF power is plotted in Fig. 4. The blue circle marks show the major values of RF power in the CCD (26, 70, 135, 200, and 244 W) in this DOE setup. When the plasma density decreases to ~ 10^9^ with a RF power reduction, the chemical reaction rate decreases due to the low ionization rate of the N_2_ plasma. Thus, a thick sheath is formed and the plasma becomes a remote mode where very few charged species reach into the substrate^35^. In this remote mode, many more N free radicals are generated than N_2_^+^ and N^+^ ions by the high ratio of electrons with low energy in the plasma. Consequently, chemical reactions for the formation of Cu nitride passivation by radicals dominate on the Cu surface rather than ion bombardment. As the plasma density increases, the plasma is converted to the direct mode and reactive ion etching (RIE) mode. These modes disturb the formation of the Cu nitride passivation because the sputtering effect caused by energetic ions accompanies it. Table 3 compares the plasma properties according to each plasma mode.

**Table 3:** Plasma properties of each plasma mode.

Plasma mode	Remote	Direct	RIE
Density (cm^−3^)	≈ 10^9^	~ 10^10^	10^10^–10^11^
Sputtering effect	Almost-free	Small	Large
Application	Surface treatment (by radical)	PECVD plasma etching	Reactive ion etching (anisotropic)
Schematic diagram			

### Electron energy distribution in the remote mode N_2_ plasma

The ionization, dissociation, and excitation reaction of neutral particles in the plasma depend on the kinetic energy of the electrons, and the kinetic energy of the electrons is proportional to the electron temperature. The electron temperature $T_{e}$ can be calculated simultaneously by Eq. (3) for the power balance equation and particle balance equation^32,36^.

3 $$ \frac{{K_{iz} \left( {T_{e} } \right)}}{{u_{B} \left( {T_{e} } \right)}} = \frac{1}{{n_{g} d_{eff} }} = \frac{1}{{n_{g} }}\frac{{A_{eff} }}{V} $$

where $K_{iz}$ is the ionization rate coefficient, gas density $n_{g} = \left( {3.3 \times 10^{19} } \right)p$ and $p$ is the working pressure. Since the plasma chamber size is fixed, we can ignore the terms $A_{eff}$ and $V$ in Eq. (3). That is, the electron temperature is only affected by the working pressure (3.5 to 8.5 mTorr), and the electron temperature exponentially decreases as the working pressure increases. This is because when the working pressure increases at a constant plasma condition, the amounts of ions and electrons increase, and the heat energy absorbed per electron decreases.

**Figure 5:**
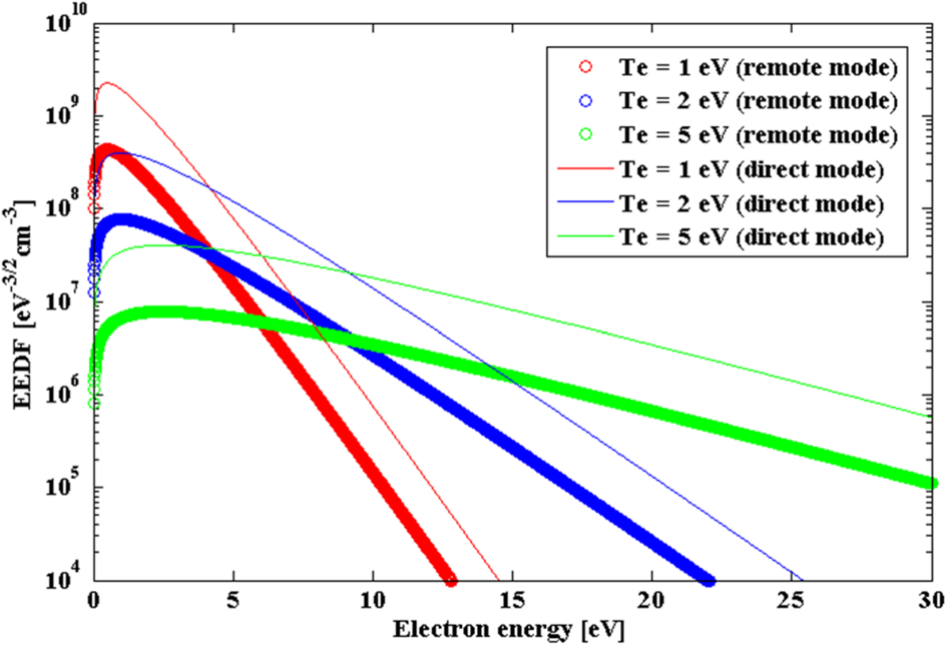
Electron energy distribution function (EEDF) with electron temperature and the plasma mode.

As the electron temperature along with the plasma density greatly affects the electron energy distribution function (EEDF), we can infer the chemical reactions occurring in the plasma through the EEDF. If we assume that the EEDF follows the Maxwell distribution, the equation can be written as^32,37,38^

4 $$ f\left( \varepsilon \right) = \frac{{2n_{e} }}{\sqrt \pi }\frac{1}{{(T_{e} )^{{{3 \mathord{\left/ {\vphantom {3 2}} \right. \kern-\nulldelimiterspace} 2}}} }}\sqrt \varepsilon e^{{{{ - \varepsilon } \mathord{\left/ {\vphantom {{ - \varepsilon } {T_{e} }}} \right. \kern-\nulldelimiterspace} {T_{e} }}}} $$

where $\varepsilon$ is the electron energy and $n_{e}$ is the electron density, which is considered to be equal to the plasma density. Figure 5 shows the constructed EEDF and it demonstrates the electron energy distributions with the electron temperature and the plasma mode. The lower electron temperature, the higher distribution of electrons with lower energy, and the reactive species in the plasma significantly decrease in the remote mode with a low plasma density.

If the kinetic energy of electrons in the plasma is insufficient, excitation or dissociation occurs more than ionization. In our experiments, most electrons in the plasma had low electron energy ($T_{e}$ < 2.4 eV) because a significant amount of energy was consumed as collisional energy for ionization or dissociation of N_2_ molecules. Therefore, the unstable state of the N free radicals generated by low-energy electron collision reached the Cu surface, and metastable Cu nitride passivation in the form of Cu_4_N was produced rather than Cu_3_N. Table 4 lists the energy required for dissociation and ionization of Ar, N_2_, and Cu^36,39^.

**Table 4:** Main reactions for each element in the plasma and required electron energy^36,39^.

Element	Reactions in the plasma	Electron energy (eV)
Ar	Ar (g) + e^−^ → Ar^+^ (g) + 2e^−^	15.8
N_2_	N_2_ (g) + e^−^ → 2 N (g) + e^−^	9.8
	N_2_ (g) + e^−^ → N_2_^+^ (g) + 2e^−^ (g)	15.3
	N_2_ (g) + e^−^ → N^+^ (g) + N (g) + 2e^−^	24.3
Cu	Ar^+^ (g) + Cu (s) → Ar^+^ (g) + Cu^+^ (g) + e^−^	11.24

### The mechanism of the Cu_4_N formation and Cu–Cu bonding

**Figure 6:**
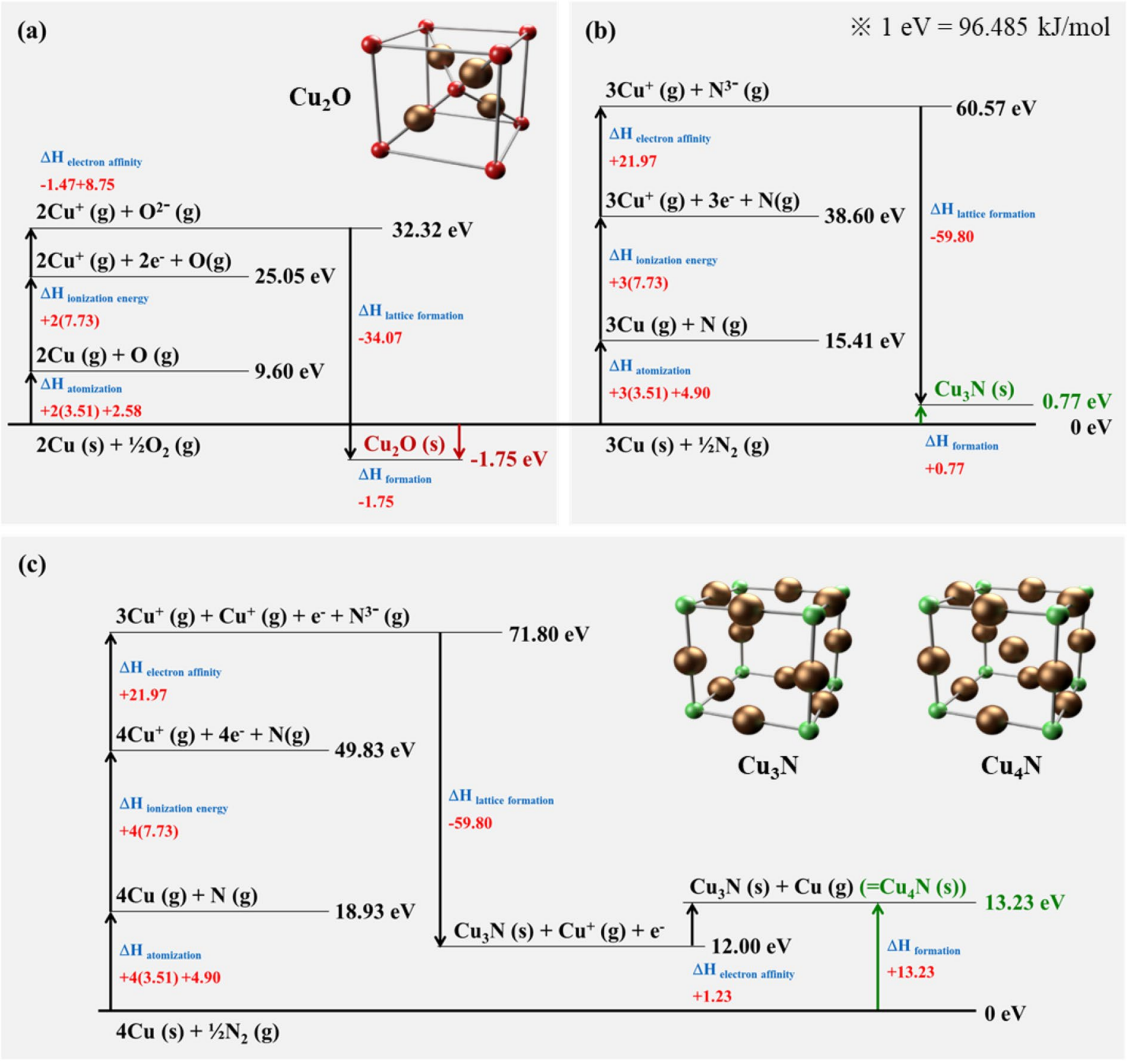
The standard enthalpy of formation according to the Born-Haber cycle (1 eV = 96.485 kJ/mol) (**a**) Cu_2_O, (**b**) Cu_3_N, and (**c**) Cu_4_N.

To study the mechanism of the Cu_4_N formation, we calculated the standard enthalpy of formation. The standard enthalpy of formation of Cu_4_N has not been reported yet, so we derived it according to the Born-Haber cycle using the enthalpy of Cu_2_O and Cu_3_N from the literature^40,41^. The standard enthalpies of formation for several Cu compounds (Cu_2_O, Cu_3_N, and Cu_4_N) are calculated in Fig. 6a–c. The equation for the standard enthalpy of formation is expressed as

5 $$ \Delta H_{atomization} + \Delta H_{ionization\;energy} + \Delta H_{electron\;affinity} + \Delta H_{lattice\;formation} = \Delta H_{formation} $$

As demonstrated in Fig. 6b, the mechanism of Cu_3_N formation is explained as follows. In order for solid Cu to be ionized, first the metallic bond must be broken and then it becomes a Cu atom (Cu (s) → Cu (g)), and the atomization energy of Cu is 3.51 eV. The strong triple bond of the N_2_ molecule is also broken to produce a N free radical (N_2_ (g) → 2 N (g)), which requires 9.8 eV of dissociation energy. With the first ionization energy of 7.73 eV, the Cu atom removes a valence electron and then becomes a Cu^+^ ion. At the same time, the neighboring N free radical that reached the Cu surface obtained three electrons, one per Cu and then became a N^3−^ ion (N (g) → N^3−^ (g)). Finally, three positive Cu ions and a negative N ion formed Cu_3_N through ion bonding with an exothermic reaction, and the standard enthalpy of formation of Cu_3_N reported by M. Akira is 0.77 eV^41^. Here, the total energy of the entire chemical reaction is conserved, so the lattice formation enthalpy is − 59.80 eV.

**Figure 7:**
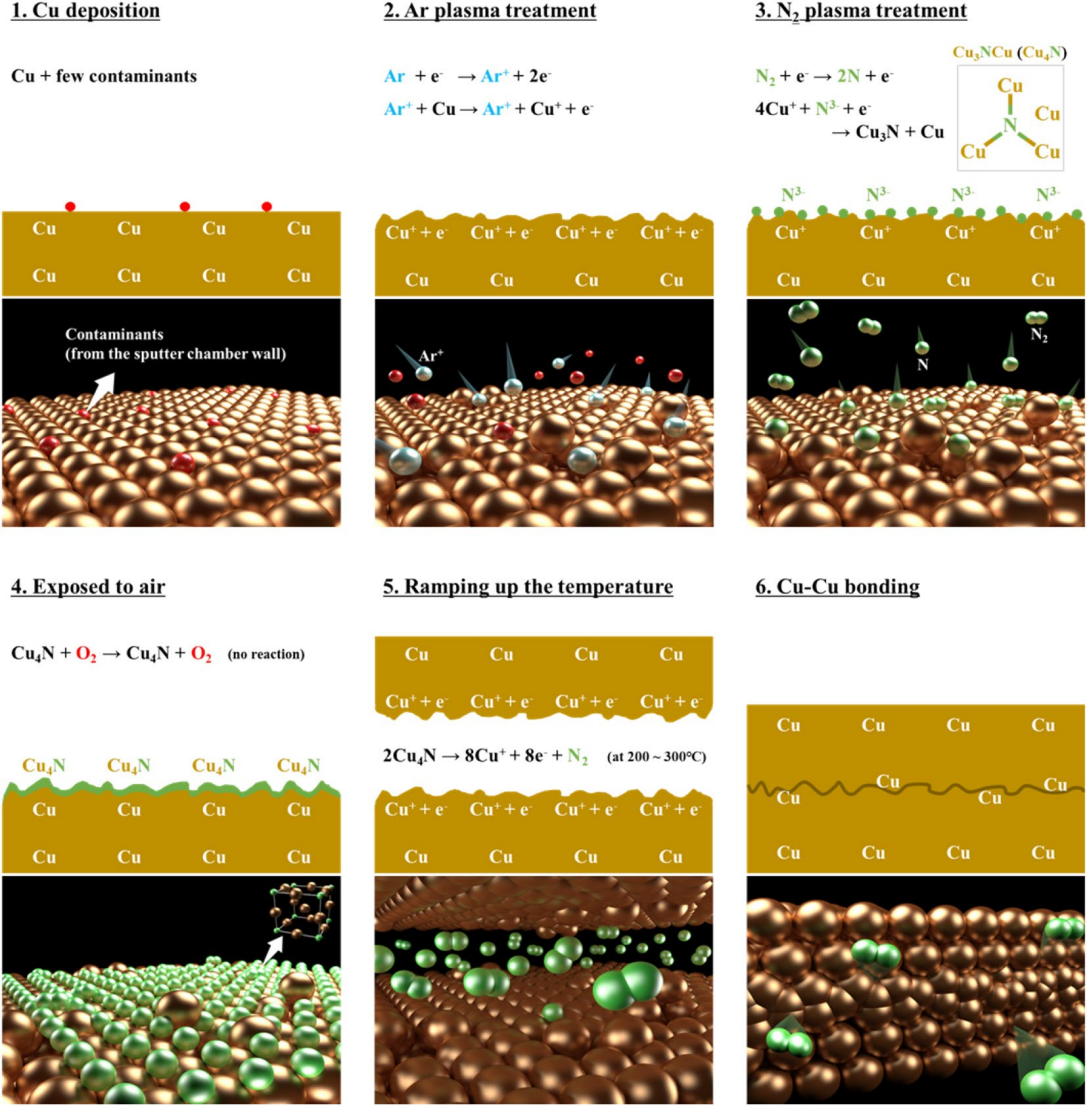
Low temperature Cu–Cu bonding mechanism using an anti-oxidant Cu layer by remote mode N_2_ plasma in the two-step Ar/N_2_ plasma process, including the formation and decomposition of Cu nitride passivation.

The mechanism of Cu_4_N formation can be explained in the same way as shown in Fig. 6c. Cu_4_N has one more Cu atom than Cu_3_N, but the lattice formation enthalpy of Cu_4_N is assumed to be the same as Cu_3_N because the Cu atom is interstitially doped into the empty space of Cu_3_N, which has a cubic anti-ReO_3_ crystal structure^42^. Therefore, the standard enthalpy of formation of Cu_4_N is 13.23 eV, which is quite high compared to Cu_2_O (− 1.75 eV) as shown in Fig. 6a. Cu_2_O has a negative enthalpy, so it is chemically stable and hard to be decomposed. It makes Cu–Cu bonding difficult at low temperature because the Cu_2_O prevents the diffusion of Cu at the bonding interface. However, when the Cu_4_N is in a metastable state, it can be decomposed at a lower temperature than the bonding temperature, which results in pure Cu–Cu bonding. Hence, this mechanism provides spontaneous Cu–Cu bonding at low temperature. The entire bonding process and mechanism, including the formation and decomposition of Cu nitride passivation, are shown in Fig. 7.

### The evaluation of the bonded interface of anti-oxidant Cu–Cu bonding

**Figure 8:**
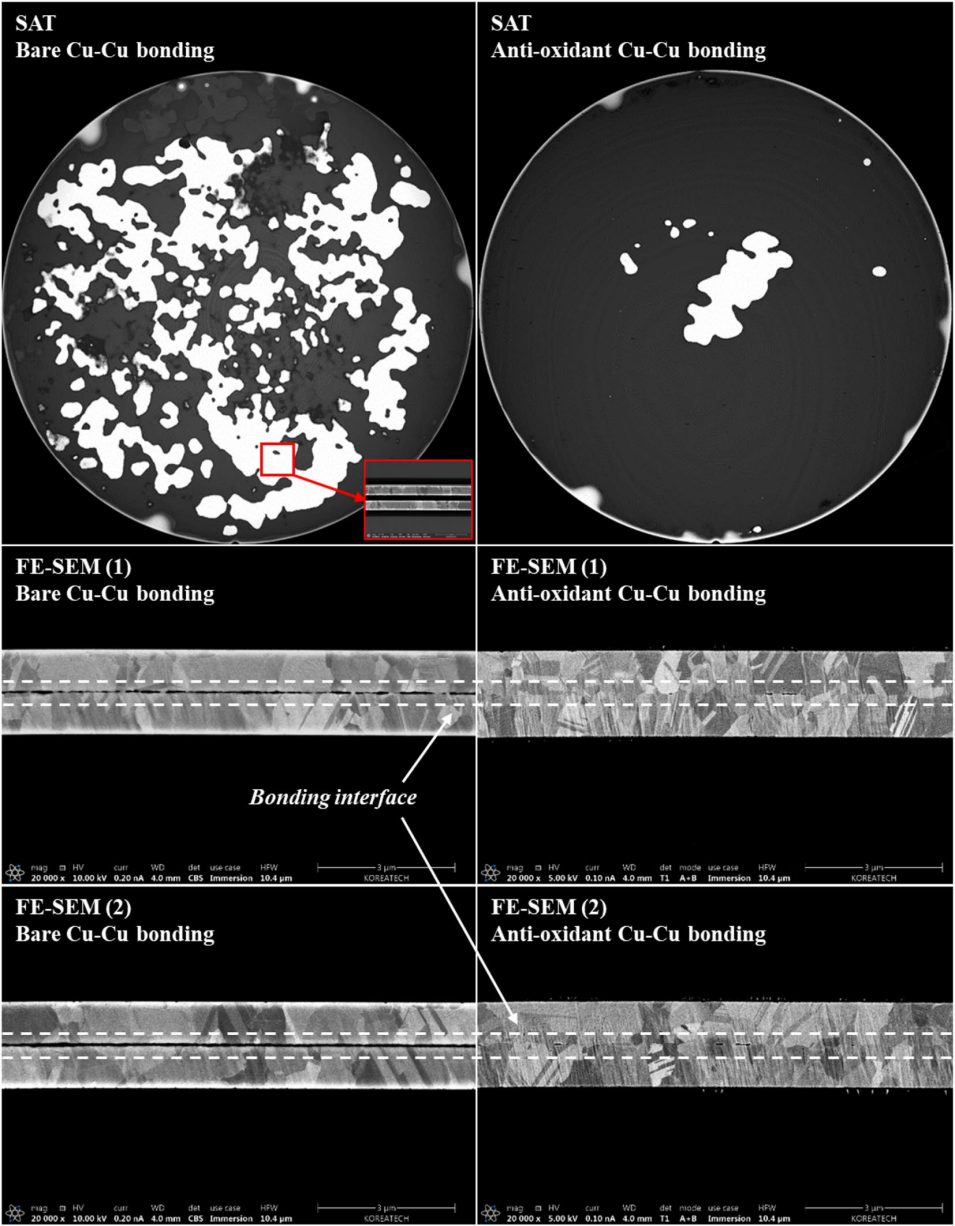
SAT and FE-SEM evaluation of the Cu–Cu interface bonded at 300 °C: (left) bonding with non-plasma treated Cu wafers and (right) bonding with two-step plasma treated Cu wafers.

A pair of Cu wafers of two types were bonded at 300 °C under 700 kPa for 1 h using a thermo-compression bonding method. Figure 8 shows the SAT and FE-SEM images for both the bare Cu–Cu bonding using non-plasma treated Cu and the anti-oxidant Cu–Cu bonding using remote mode N_2_ plasma treated Cu in the two-step Ar/N_2_ plasma process. In the SAT analysis, note that any unbonded voids appear as white color due to the reflection of the ultrasonic signal. As shown in Fig. 8 (left), bare Cu–Cu bonding has an extremely poor bonded interface, which is due to Cu surface oxidation; moreover, the cross-sectional image of the bonded interface in the FE-SEM image clearly shows an unbonded interface. On the other hand, anti-oxidant Cu–Cu bonding demonstrates an improved bonding quality in most of the wafer except for the central region. This unbonded area appears to be caused by trapped N_2_ gas when the Cu nitride passivation on the entire wafer is thermally decomposed during the bonding process. In addition, there was Cu atomic diffusion with new grains across the bonded interface. Considering that this is whole Cu blanket wafer bonding without a CMP process, it demonstrates a significant improvement and a high potential for low temperature Cu–Cu bonding.

## Conclusion

A low temperature Cu–Cu bonding process is essential in advanced 3D device stacking technologies to protect the devices from thermal damage, and the key point of Cu–Cu bonding is preventing oxidation on a Cu surface especially for die-to-wafer stacking. In this study, we protected the Cu surface from oxidation by the formation of Cu nitride passivation using a two-step Ar/N_2_ plasma treatment. After the Cu surface activation by Ar plasma pre-treatment, N free radicals in the N_2_ plasma treatment reacted with the Cu surface to form Cu nitride passivation. According to a chemical state analysis of the two-step Ar/N_2_ plasma treated Cu surface by XPS and a calculation of the standard enthalpy of formation, the Cu nitride layer effectively prevented the formation of native Cu oxide and it promotes spontaneous Cu–Cu bonding.

We statistically analyzed the effect of N_2_ plasma variables on the formation of Cu nitride passivation by the RSM using a CCD in DOE methodology. Among the N_2_ plasma variables, RF power was the most effective factor for the formation of Cu nitride passivation, and a lower RF power generated the remote mode N_2_ plasma. An anti-oxidant Cu layer was obtained in the remote mode N_2_ plasma with low electron temperature and low plasma density. A pair of anti-oxidant Cu wafers were bonded at 300 °C, and the bonding quality was significantly improved over bare Cu–Cu bonding.

In the future, we will optimize the N_2_ plasma treatment conditions to provide improved Cu–Cu bonding quality at a lower temperature and perform electrical and mechanical evaluations.

## References

[1] Li Y, Goyal D (2017). 3D microelectronic packaging: from fundamentals to applications. Springer Ser. Adv. Microelectron..

[2] List, R. S., Webb, C. & Kim, S. E. 3D wafer stacking technology. In *Proceedings of the Advanced Metallization Conference*. 29–36 (2002).

[3] Ko C, Chen K (2012). Low temperature bonding technology for 3D integration. Microelectron. Reliab..

[4] Morrow, P., et al. Wafer level 3D interconnects via Cu bonding. In *Proceedings of the Advanced Metallization Conference.* 125–130 (2004).

[5] Panigrahy AK, Chen K (2018). Low temperature Cu–Cu bonding technology in three-dimensional integration: an extensive review. J. Electron. Packag..

[6] Das S, Chandrakasan AP, Reif R (2004). Calibration of Rent's rule models for three-dimensional integrated circuits. IEEE VLSL Syst..

[7] Chen, F. C., Chen, M. F., Chiou, W. C. & Yu, D. H. System on integrated chips (SoIC^TM^) for 3D heterogeneous integration. In *Proceedings of the IEEE 69th Electronic Components and Technology Conference (ECTC)*. 594–599 (2019).

[8] Hilton A, Temple DS (2016). Wafer-level vacuum packaging of smart sensors. MDPI Sens..

[9] Chua SL, Tan CS (2016). Cu-Cu die to die surface activated bonding in atmospheric environment using Ar and Ar/N_2_ plasma. Electrochem. Soc..

[10] Juang JY, Lu CL, Chen KJ, Chen CC, Hsu PN, Chen C, Tu KN (2018). Copper-to-copper direct bonding on highly (111)-oriented nanotwinned copper in no-vacuum ambient. Sci. Rep..

[11] Liu J, Mou Y, Peng Y, Sun Q, Chen M (2019). Novel Cu-Ag composite nanoparticle paste for low temperature bonding. Mater. Lett..

[12] Lim DF, Wei J, Leong KC, Tan CS (2013). Cu passivation for enhanced low temperature (≤ 300 °C) bonding in 3D integration. Microelectron. Eng..

[13] Takagi H, Kikuchi K, Maeda R, Chung TR, Suga T (1996). Surface activated bonding of silicon wafers at room temperature. Appl. Phys. Lett..

[14] He R, Fujino M, Yamauchi A, Wang Y, Suga T (2016). Combined surface activated bonding technique for low-temperature Cu/dielectric hybrid bonding. ECS J. Solid State Sci. Technol..

[15] Jangam, S., et al. Fine-Pitch (≤ 10 μm) direct Cu–Cu interconnects using in-situ formic acid vapor treatment. In *Proceedings of the IEEE 69th Electronic Components and Technology Conference (ECTC)*. 620–627 (2019).

[16] Fan J, Lim DF, Tan CS (2013). Effects of surface treatment on the bonding quality of wafer-level Cu-to-Cu thermo-compression bonding for 3D integration. J. Micromech. Microeng..

[17] Tan CS, Lim DF, Ang XF, Wei J, Leong KC (2012). Low temperature Cu-Cu thermo-compression bonding with temporary passivation of self-assembled monolayer and its bond strength enhancement. Microelectron. Reliab..

[18] Peng, L., Li, H. Y., Lim, D. F., Gao, S. & Tan, C. S. Thermal reliability of fine pitch Cu-Cu bonding with self assembled monolayer (SAM) passivation for wafer-on-wafer 3D-Stacking. In *Proceedings of the IEEE 61th Electronic Components and Technology Conference (ECTC)*. 22–26 (2011).

[19] Panigrahi AK, Ghosh T, Vanjari SRK, Gingh SG (2017). Oxidation resistive, CMOS compatible copper-based alloy ultrathin films as a superior passivation mechanism for achieving 150 °C Cu-Cu wafer on wafer thermocompression bonding. IEEE Trans. Electron Dev..

[20] Bonam S, Panigrahi AK, Kumar CH, Vanjari SRK, Singh SG (2019). Interface and reliability analysis of Au-passivated Cu–Cu fine-pitch thermocompression bonding for 3-D IC applications. IEEE Trans. Compon. Packag. Manuf. Technol..

[21] Sun L, Chen MH, Zhang L (2019). Microstructure evolution and grain orientation of IMC in Cu-Sn TLP bonding solder joints. J. Alloys Compd..

[22] Wang J, Wang Q, Wu Z, Wang D, Cai J (2016). Solid-state-diffusion bonding for wafer-level fine-pitch Cu/Sn/Cu interconnect in 3-D integration. IEEE Trans. Compon. Packag. Manuf. Technol..

[23] Gao, G., et al. Development of low temperature direct bond interconnect technology for die-to-wafer and die-to-die applications-stacking, yield improvement, reliability assessment. In *Proceedings of the IEEE 2018 International Wafer Level Packaging Conference (IWLPC)*. 1–7 (2018).

[24] Enquist, P., Fountain, G., Petteway, C., Hollingsworth, A. & Grady, H. Low cost of ownership scalable copper Direct Bond Interconnect 3D IC technology for three dimensional integrated circuit applications. In *Proceedings of the IEEE 2009 International Conference on 3D System Integration*. 1–6 (2009).

[25] Allen TT (2019). Introduction to Engineering Statistics and Lean Six Sigma: Statistical Quality Control and Design of Experiments and SYSTEMS.

[26] Omulo G, Banadda N, Kabenge I, Seay J (2019). Optimizing slow pyrolysis of banana peels wastes using response surface methodology. Environ. Eng. Res..

[27] Park H, Kim SE (2020). Two-step plasma treatment on copper surface for low-temperature Cu thermo-compression bonding. IEEE Trans. Compon. Packag. Manuf. Technol..

[28] Wagner, C. D. Handbook of x-ray photoelectron spectroscopy: A reference book of standard data for use in x-ray photoelectron spectroscopy. In *Perkin-Elmer* 82–83 (1979).

[29] Kim JP, Park ES, Hong TE, Bae JS, Ha MG, Jin JS, Jeong ED, Hong KS (2012). Electric properties and chemical bonding states of pn-junction p-CuO/n-Si by sol-gel method. J. Ceram. Process. Res..

[30] Siriwardane RV, Poston JA (1993). Characterization of copper oxides, iron oxides, and zinc copper ferrite desulfurization sorbents by X-ray photoelectron spectroscopy and scanning electron microscopy. Appl. Surf. Sci..

[31] Venkata Subba Reddy K, Uthanna S (2007). Effect of sputtering power on the physical properties of Cu_3_N films formed by DC magnetron sputtering. Synth. React. Inorg. Met. Org. Nano-Met Chem..

[32] Lieberman MA, Lichtenberg AJ (2005). Principles of Plasma Discharges and Materials Processing.

[33] Balcon N, Aanesland A, Boswell R (2007). Pulsed RF discharges, glow and filamentary mode at atmospheric pressure in argon. Plasma Sources Sci. Technol..

[34] Berenguer C, Katsonis K (2012). Plasma reactors and plasma thrusters modeling by Ar complete global models. Int. J. Aerosp. Eng..

[35] Hanyaloglu BF, Aydil ES (1998). Low temperature plasma deposition of silicon nitride from silane and nitrogen plasmas. J. Vac. Sci. Technol. Vac. Surf. Films.

[36] El-Sayed NM (2018). Electron collision rates and energy loss in argon and nitrogen glow discharge plasma. Arab. J. Nucl. Sci. Appl..

[37] Khalilpour H, Foroutan G (2020). The effects of electron energy distribution function on the plasma sheath structure in the presence of charged nanoparticles. J. Plasma Phys..

[38] Ramamurthi B, Economou DJ, Kaganovich ID (2003). Effect of electron energy distribution function on power deposition and plasma density in an inductively coupled discharge at very low pressures. Plasma Sources Sci. Technol..

[39] Hänninen T (2018). Silicon nitride based coatings grown by reactive magnetron sputtering. Linköping Stud. Sci. Technol. Diss..

[40] Wagman DD (1982). The NBS Tables of chemical thermodynamic properties: selected values for inorganic and C_1_ and C_2_ organic substances in SI units. J. Phys. Chem. Ref. Data.

[41] Miura A, Takei T, Kumada N (2014). Synthesis of Cu_3_N from CuO and NaNH_2_. J. Asian Ceram. Soc..

[42] Lu N, Ji A, Cao Z (2013). Nearly constant electrical resistance over large temperature range in Cu_3_NM_x_ (M = Cu, Ag, Au) compounds. Sci. Rep..

